# Adverse Outcomes after Non-Chest Surgeries in Patients with Pulmonary Tuberculosis: A Nationwide Study

**DOI:** 10.1371/journal.pone.0133064

**Published:** 2015-07-14

**Authors:** Chi-Chen Ke, Chao-Shun Lin, Chun-Chieh Yeh, Chi-Li Chung, Chih-Jen Hung, Chien-Chang Liao, Ta-Liang Chen

**Affiliations:** 1 Department of Anesthesiology, Taichung Veterans General Hospital, Taichung, Taiwan; 2 Department of Anesthesiology, Taipei Medical University Hospital, Taipei, Taiwan; 3 Health Policy Research Center, Taipei Medical University Hospital, Taipei, Taiwan; 4 School of Medicine, Taipei Medical University, Taipei, Taiwan; 5 Department of Surgery, China Medical University Hospital, Taichung, Taiwan; 6 Department of Surgery, University of Illinois, Chicago, United States of America; 7 Division of Pulmonary Medicine, Department of Internal Medicine, Taipei Medical University Hospital, Taipei, Taiwan; 8 School of Chinese Medicine, China Medical University, Taichung, Taiwan; Universidad Nacional de La Plata., ARGENTINA

## Abstract

**Background:**

The association between pulmonary tuberculosis (TB) and postoperative outcomes remains unknown. This study investigated outcomes following non-chest surgeries in patients with previous pulmonary TB.

**Methods:**

Using Taiwan’s National Health Insurance Research Database, we analyzed 6911 patients (aged ≥ 20 years) with preoperative diagnosis of pulmonary TB and 6911 propensity score-matched controls receiving non-chest surgeries in 2008–2010. Postoperative outcomes were compared between patients with or without pulmonary TB by calculating adjusted odds ratios (ORs) and 95% confidence intervals (CIs) in the multivariate logistic regressions.

**Results:**

Surgical patients with pulmonary TB had a significantly higher postoperative complication rates than controls, including septicemia, pneumonia, acute renal failure, deep wound infection, overall complications, and 30-day postoperative mortality (OR 1.41; 95% CI 1.07–1.86). The ORs of patients with low-income status were as high as 2.27 (95% CI 1.03–5.03). Preoperative use of TB drugs and TB-related medical expenditure also associated with higher postoperative mortality among surgical patients with pulmonary TB.

**Conclusions:**

Surgical patients with pulmonary TB have significantly increased risks of postoperative complications and mortality after non-chest surgeries. This study suggests the need to improve postoperative care for surgical patients with pulmonary TB.

## Introduction

Pulmonary tuberculosis (TB) remains a major health problem causing high mortality and morbidity worldwide [1]. The World Health Organization estimated that 8.6 million people were infected with TB in 2012 and that the disease caused 1.3 million deaths in that year [1]. Over 90% of pulmonary TB cases were reported in developing countries, and most occurred in Asia [2]. More than US$ 7 billion annually is needed for a full response to the pulmonary TB epidemic [1]. Risk factors associated with mortality in TB patients include advanced age, male gender, malnutrition, diabetes mellitus, chronic renal disease, silicosis, prolonged corticosteroid use, substance abuse, human immunodeficiency virus infection, and receiving transplant surgery [3–11].

Comorbidities and risk factors for surgical patients with pulmonary TB are not completely understood. Pulmonary TB may co-exist with several medical conditions, such as chronic lung disease [12], lung cancer [13], stroke [14], pneumonia [15], and septic shock [16]; these were found to be significant predictors of mortality and might substantially influence patient outcomes [17,18]. Previous studies on postoperative complications and mortality of TB patients were limited to thoracic surgery, and the comprehensive features of perioperative adverse outcomes in surgical patients with pulmonary TB were unknown [19].

Using reimbursement claims from Taiwan’s National Health Insurance Research Database, we conducted a nationwide retrospective cohort study to investigate adverse events after non-chest surgeries among patients with pulmonary TB.

## Methods

### Source of Data

This retrospective cohort study used the reimbursement claims of Taiwan’s National Health Insurance. This program that was launched in March 1995 and covers more than 99% of the 22.6 million residents of Taiwan. As described in detail previously [20–22], the National Health Research Institutes established the National Health Insurance Research Database to record all beneficiaries’ inpatient and outpatient medical services, including simple demographics, primary and secondary diagnoses, procedures, prescriptions, and medical expenditures. Research articles based on Taiwan’s National Health Insurance Research Database have been accepted in distinguished scientific journals worldwide [20–22].

### Ethical Approval

As described elsewhere [20–22], insurance reimbursement claims from this database are available for academics to access by application. In order to protect personal privacy and to comply with the Helsinki Declaration, the electronic database with patients’ identifications was decoded and scrambled for research access. Because of this privacy protection, informed consent was not required. The study was evaluated and approved by Taiwan’s National Health Research Institutes (NHIRD-103-121) [20–22].

### Study Population

We examined medical claims and identified 7077 patients more than 20 years old with preoperative pulmonary TB from 1,499,745 patients who underwent major inpatient non-chest surgeries between 2008 and 2010 in Taiwan. These surgeries required hospitalization for more than one day and general, epidural, or spinal anesthesia. Previous studies considered patients with pulmonary TB as those who had made two visits for outpatient care for principal or secondary TB diagnoses [23]. To identify patients with pulmonary TB more strictly and to avoid those with diagnostic errors, this study required at least two visits for outpatient care or one visit for hospital admission attributable to pulmonary TB with a principal diagnosis within the 24-month preoperative period. Using a propensity score-matched pair procedure, we randomly selected surgical patients without pulmonary TB from the database to match each surgical patient with pulmonary TB by sex, age, low income, coexisting medical conditions, types of surgery and anesthesia, and whether the surgery was performed in a teaching hospital.

### Measures and Definitions

According to National Health Insurance regulations, patients’ income status was identified by defining those qualifying for waived medical co-payment. Also recorded were whether the surgery was performed in a teaching hospital and types of non-chest surgeries and anesthesia. We defined pulmonary TB (ICD-9-CM 011), related coexisting diseases during the 24-month period before surgery, and complications after surgery according to the *International Classification of Diseases*, *9* ^*th*^ *Revision*, *Clinical Modification* (ICD-9-CM). These coexisting medical conditions included diabetes (ICD-9-CM 250), mental disorders (ICD-9-CM 290–319), ischemic heart disease (ICD-9-CM 410–414), congestive heart failure (ICD-9-CM 428), chronic obstructive pulmonary disease (ICD-9-CM 490–496), liver cirrhosis (ICD-9-CM 571), peripheral vascular disease (ICD-9-CM 443), anemia (ICD-9-CM 280–285), atrial fibrillation (ICD-9-CM 427.31), and Parkinson’s disease (ICD-9-CM 332) [24]. Renal dialysis was defined by administration code (D8, D9). In-hospital 30-day mortality after the index surgery was considered the study’s primary outcome. Eight major postoperative complications after the index surgery were analyzed as secondary outcomes, including septicemia (ICD-9-CM 038 and 998.5), pneumonia (ICD-9-CM 480–486), stroke (ICD-9-CM 430–438), acute renal failure (ICD-9-CM 584), deep wound infection (ICD-9-CM 958.3), acute myocardial infarction (ICD-9-CM 410), postoperative bleeding (ICD-9-CM 998.0, 998.1 and 998.2), and pulmonary embolism (ICD-9-CM 415) [20–22].

### Statistical Analysis

To reduce confounding errors, we developed a non-parsimonious multivariable logistic regression model to estimate a propensity score for preoperative pulmonary TB, irrespective of outcome. Covariates in this model included age, sex, low income, diabetes, mental disorders, ischemic heart disease, renal dialysis, congestive heart failure, chronic obstructive pulmonary disease, liver cirrhosis, peripheral vascular disease, anemia, atrial fibrillation, Parkinson’s disease, human immunodeficiency virus infection/ acquired immune deficiency syndrome, teaching hospital, types of surgery, types of anesthesia, previous organ transplantation, emergent surgery, and use of immunosuppressants (such as azathioprine, cyclosporin, dexamethasone, everolimus, hydrocortisone, methotrexate, mycophenolate, prednisone, tacrolimus, and thalidomide). We used a structured iterative approach to refine this model to achieve covariate balance within the matched pairs. The covariate balance was measured in the chi-square tests, and a *P* value lower than 0.05 suggested significant imbalance. We matched patients with pulmonary TB to patients without pulmonary TB using a greedy-matching algorithm with a width of 0.2 SD of the log odds of the estimated propensity score.

We performed multivariate logistic regressions to calculate adjusted odds ratios (ORs) and 95% confidence intervals (CIs) of postoperative complications and mortality associated with preoperative pulmonary TB after adjustment for age, sex, low income, diabetes, mental disorders, ischemic heart disease, renal dialysis, congestive heart failure, chronic obstructive pulmonary disease, liver cirrhosis, peripheral vascular disease, anemia, atrial fibrillation, Parkinson’s disease, human immunodeficiency virus infection/ acquired immune deficiency syndrome, teaching hospital, types of surgery, types of anesthesia, previous organ transplantation, emergent surgery, and use of immunosuppressants. The joint effects of coexisting medical conditions on pulmonary TB associated with 30-day postoperative mortality were assessed in the multivariate logistic regressions with calculated adjusted ORs and 95% CIs. The impacts of low-income status and use of anti-TB medication on postoperative mortality were also evaluated in the multivariate logistic regressions.

## Results

Table 1 shows the demographic characteristics of patients with and without pulmonary TB who underwent non-chest surgeries. After propensity score matching, there were no statistical differences between these two groups analyzed by age, sex, low income, diabetes, mental disorders, ischemic heart disease, renal dialysis, congestive heart failure, chronic obstructive pulmonary disease, liver cirrhosis, peripheral vascular disease, anemia, atrial fibrillation, Parkinson’s disease, human immunodeficiency virus infection/ acquired immune deficiency syndrome, teaching hospital, types of surgery, types of anesthesia, previous organ transplantation, emergent surgery, and use of immunosuppression.

**Table 1:** Characteristics of Surgical Patients with and without Pulmonary Tuberculosis.

	No TB (N = 6911)		TB (N = 6911)		
	n	(%)	n	(%)	p-value
Age, years					1.0000
20–29	204	(3.0)	204	(3.0)	
30–39	333	(4.8)	333	(4.8)	
40–49	649	(9.4)	649	(9.4)	
50–59	1050	(15.2)	1050	(15.2)	
60–69	1208	(17.5)	1208	(17.5)	
70–79	1957	(28.3)	1957	(28.3)	
≥80	1510	(21.9)	1510	(21.9)	
Sex					1.0000
Female	2029	(29.4)	2029	(29.4)	
Male	4882	(70.6)	4882	(70.6)	
Low income	300	(4.3)	300	(4.3)	1.0000
Coexisting medical conditions					
COPD	2523	(36.5)	2523	(36.5)	1.0000
Mental disorders	1601	(23.2)	1601	(23.2)	1.0000
Diabetes	1361	(19.7)	1361	(19.7)	1.0000
Ischemic heart disease	905	(13.1)	905	(13.1)	1.0000
Liver cirrhosis	277	(4.0)	277	(4.0)	1.0000
Congestive heart failure	267	(3.9)	267	(3.9)	1.0000
Renal dialysis	234	(3.4)	234	(3.4)	1.0000
Anemia	188	(2.7)	188	(2.7)	1.0000
Parkinson’s disease	140	(2.0)	140	(2.0)	1.0000
Peripheral vascular disease	38	(0.6)	38	(0.6)	1.0000
Atrial fibrillation	33	(0.5)	33	(0.5)	1.0000
HIV infection or AIDS	73	(1.1)	73	(1.1)	1.0000
Types of surgery					1.0000
Musculoskeletal	2129	(30.8)	2129	(30.8)	
Digestive	1829	(26.5)	1829	(26.5)	
Neurosurgery	798	(11.6)	798	(11.6)	
Kidney, Ureter, Bladder	644	(9.3)	644	(9.3)	
Cardiovascular	396	(5.7)	396	(5.7)	
Skin	154	(2.2)	154	(2.2)	
Delivery, CS, Abortion	87	(1.3)	87	(1.3)	
Eye	79	(1.1)	79	(1.1)	
Breast	39	(0.6)	39	(0.6)	
Others	756	(10.9)	756	(10.9)	
Types of anesthesia					1.0000
General	4915	(71.1)	4915	(71.1)	
Epidural or Spinal	1996	(28.9)	1996	(28.9)	
Previous organ transplantation	2	(0.03)	2	(0.03)	1.0000
Emergent surgery	1105	(16.0)	1105	(16.0)	1.0000
Use of immunosuppressants*	2745	(39.7)	2745	(39.7)	1.0000

COPD, chronic obstructive pulmonary disease; TB, pulmonary tuberculosis.

*including azathioprine, cyclosporin, dexamethasone, everolimus, hydrocortisone, methotrexate, mycophenolate, prednisone, tacrolimus, and thalidomide

Compared with people without pulmonary TB (Table 2), patients with pulmonary TB showed higher risk of postoperative complications, including septicemia (OR 1.39; 95% CI 1.22–1.59), pneumonia (OR 1.55; 95% CI 1.33–1.81), pulmonary embolism (OR 2.83; 95% CI 1.01–7.91), and overall complications (OR 1.35; 95% CI 1.23–1.48). Preoperative pulmonary TB was associated with an increase in 30-day postoperative mortality (OR 1.41; 95% CI 1.07–1.86). Prolonged length of hospital stay, stay in intensive care unit, and increased medical expenditure were all significantly associated with preoperative pulmonary TB, with respective ORs of 1.35 (95% CI 1.24–1.47), 1.34 (95% CI 1.24–1.44), and 1.23 (95% CI 1.12–1.34).

**Table 2:** Risk of Postoperative Complications and Mortality for Surgical Patients with Preoperative Pulmonary Tuberculosis.

	No TB		TB		OR	(95% CI)^a^
Postoperative complications	n	(%)	n	(%)		
Septicemia	452	(6.5)	614	(8.9)	1.39	(1.22–1.59)
Pneumonia	288	(4.2)	437	(6.3)	1.55	(1.33–1.81)
Stroke	264	(3.8)	269	(3.9)	1.02	(0.85–1.21)
Acute renal failure	115	(1.7)	130	(1.9)	1.12	(0.86–1.46)
Deep wound infection	46	(0.7)	50	(0.7)	1.06	(0.71–1.59)
Acute myocardial infarction	44	(0.6)	40	(0.6)	0.89	(0.57–1.38)
Postoperative bleeding	61	(0.9)	45	(0.7)	0.73	(0.50–1.08)
Pulmonary embolism	5	(0.1)	14	(0.2)	2.83	(1.01–7.91)
Any of the above	1034	(15.0)	1309	(18.9)	1.35	(1.23–1.48)
30-day in-hospital mortality	92	(1.3)	126	(1.8)	1.41	(1.07–1.86)
ICU stay	2481	(35.9)	2873	(41.6)	1.34	(1.24–1.44)
Prolonged length of hospital stay	1192	(17.3)	1500	(21.7)	1.35	(1.24–1.47)
Elevated medical expenditure	1275	(18.5)	1488	(21.5)	1.23	(1.12–1.34)
Medical expenditure, USD^b^	3072±5639		3484±5715		p<0.0001	
Length of hospital stay, days^b^	9.6±12.8		11.7±17.8		p<0.0001	

CI, confidence interval; ICU, intensive care unit; OR, odds ratio; TB, pulmonary tuberculosis.

^a^Adjusted for age, sex, low income, urbanization, types of anesthesia, types of surgery, coexisting diseases, organ transplantation, steriod use and emergency operation.

^b^Mean±SD

Compared to people without pulmonary TB, patients with pulmonary TB who had certain coexisting medical conditions were at higher risk of postoperative mortality (Table 3). These coexisting conditions included renal dialysis (OR 3.74; 95% CI 1.93–7.26), congestive heart failure (OR 2.61; 95% CI 1.38–4.92), cancer (OR 1.77; 95% CI 1.16–2.69) and anemia (OR 2.56; 95% CI 1.15–5.70). Several other preoperative coexisting medical conditions had no significant impact on postoperative mortality.

**Table 3:** The Impact of Coexisting Medical Conditions on 30-day Postoperative Mortality for Surgical Patients with Preoperative Pulmonary Tuberculosis.

	30-day in-hospital mortality				
	n	Deaths	Mortality, %	OR	(95% CI)^a^
Non-PTB controls	6911	92	1.3	1.00	(reference)
PTB patients with diabetes	1361	19	1.4	0.99	(0.60–1.64)
PTB patients with mental disorders	1601	30	1.9	1.39	(0.91–2.13)
PTB patients with ischemic heart disease	905	16	1.8	0.99	(0.57–1.71)
PTB patients with renal dialysis	234	12	5.1	3.74	(1.93–7.26)
PTB patients with congestive heart failure	267	12	4.5	2.61	(1.38–4.92)
PTB patients with COPD	2523	49	1.9	1.34	(0.94–1.92)
PTB patients with liver cirrhosis	277	6	2.2	1.92	(0.82–4.50)
PTB patients with peripheral vascular disease	38	0	0.0	-	-
PTB patients with anemia	188	7	3.7	2.56	(1.15–5.70)
PTB patients with atrial fibrillation	33	0	0.0	-	-
PTB patients with Parkinson's disease	140	4	2.9	1.81	(0.65–5.09)
PTB patients with cancer	1326	31	2.3	1.77	(1.16–2.69)
PTB patients with HIV	73	0	0.0	-	-

CI, confidence interval; COPD, chronic obstructive pulmonary disease; PTB, pulmonary tuberculosis; OR, odds ratio.

^a^Adjusted for age, sex, low income, urbanization, coexisting medical conditions, types of anesthesia, types of surgery, organ transplantation, steriod use and emergency operation.

Among patients with pulmonary TB (Table 4), low-income status had a significant impact on postoperative mortality (OR 2.27; 95% CI 1.03–5.03). Preoperative use of TB-related medication also contributed to significantly higher risk of mortality after surgery in patients with pulmonary TB (OR 1.61; 95% CI 1.15–2.25). Significantly higher risk of postoperative mortality was found in people with pulmonary TB who had high expenditure on TB-related medication (OR 1.93; 95% CI 1.15–3.25). Preoperative emergency care for TB (OR 2.59; 95% CI 1.39–4.82), age ≥65 years (OR 1.52; 95% CI 1.12–2.05), and opportunistic infection (OR 3.00; 95% CI 1.35–6.67) were also important factors associated with increased risk of postoperative mortality in patients with pulmonary TB.

**Table 4:** Preoperative Characteristics of Pulmonary Tuberculosis and Risk of 30-day Postoperative Mortality.

Characteristics of PTB within preoperative 24 months		Mortality			
	n	Deaths	Mortality, %	OR	(95% CI)^a^
No PTB	6911	92	1.3	1.00	(reference)
Low income^b^					
TB without low income	6611	119	1.8	1.38	(1.05–1.82)
TB with low income	300	7	2.3	2.27	(1.03–5.03)
Use of anti-PTB treatment					
TB patients without anti-PTB treatment	4104	66	1.6	1.27	(0.92–1.76)
TB patients with anti-PTB treatment	2807	60	2.1	1.61	(1.15–2.25)
Expenditure of PTB medication					
Low and moderate	6211	108	1.7	1.35	(1.02–1.80)
Very high	700	18	2.6	1.93	(1.15–3.25)
Preoperative emergency care for PTB					
TB without emergency	6560	114	1.7	1.35	(1.02–1.78)
TB with emergency	351	12	3.4	2.59	(1.39–4.82)
The impact of age on PTB					
PTB with age of 20–64 years	2744	39	1.4	1.20	(0.82–1.77)
PTB with age of ≥65 years	4167	87	2.1	1.52	(1.12–2.05)
Opportunistic infection with PTB					
PTB without opportunistic infection	6728	119	1.8	1.37	(1.04–1.81)
PTB with opportunistic infection	183	7	3.8	3.00	(1.35–6.67)

CI, confidence interval; PTB, pulmonary tuberculosis; OR, odds ratio.

^a^Adjusted for age, sex, low income, urbanization, coexisting medical conditions, types of anesthesia, types of surgery, organ transplantation, steriod use and emergency operation.

^b^Adjusted for all variables except low income.

## Discussion

This nationwide, population-based, propensity score-matched, retrospective cohort study showed that surgical patients with pulmonary TB faced significantly increased risks of postoperative complications such as septicemia, pneumonia, acute renal failure, deep wound infection, and 30-day postoperative mortality. In addition, coexisting diseases in patients with TB were highly correlated with postoperative adverse outcomes.

We believe this is the first study to quantitatively associate preoperative pulmonary TB with increased in-hospital medical expenditure and with prolonged hospital and postoperative intensive care unit stays when receiving non-chest major surgeries.

Age, sex, and socioeconomic status were important risk factors for surgical patients with pulmonary TB [25]. Sociodemographic factors also were associated with perioperative outcomes [20,21]. After adjusting patients’ sociodemographic characteristics, our study showed that low-income status was an independent factor associated with postoperative complications and mortality in patients with pulmonary TB. Previous studies have focused on the prognostic impacts of such comorbidities as history of diabetes, mental disorders, congestive heart failure and anemia, which were proven to be independently associated with higher risks of postoperative complications and mortality [21,26–28]. After using the propensity score-matched pair procedure to reduce confounding bias, we showed that postoperative 30-day mortality and adverse outcomes increased significantly among patients with pulmonary TB when preoperative coexisting medical conditions became more complex.

Previous studies indicated that the older people, active phase, more coexisting medical diseases, lower socioeconomic status, opportunistic infection emergency care for TB and high-dose medication of TB were the factor associated with the higher incidence and mortality of TB [29–35]. However, the impact of these clinical scenarios on perioperative outcomes after surgery in TB patients remains unknown. These previous findings prompt us to investigate whether TB-related clinical scenarios were associated with complications and mortality after non-chest surgery in patients with pulmonary TB. Our results showed significant increases in postoperative mortality rates in TB patients who were elderly or had anti-TB treatment, emergency care, combined subtypes of TB, opportunistic infections, or high medical expenditure for TB within 24 months preoperatively. Older people with TB and those who had preoperative emergency care due to TB may be more likely to have poor physical status or critical complications at time of admission for surgery [18,29]. Elderly patients tend to have comorbidities and poor physiologic reserves that may cause higher mortality after operations. Patients receiving surgery are at risk of having worse postoperative outcomes due to decreased ability to maintain physiologic stability during surgery [36]. Furthermore, tuberculosis may not only affect the lungs, but also other organs. Our results implied that patients with coexisting extrapulmonary TB or with opportunistic infection combined with pulmonary TB might potentially experience more serious outcomes. Patients who developed extra-pulmonary TB and opportunistic infections were more difficult to treat due to compromised immune function [30,37]. Early recognition of associated medical conditions is important to reduce postoperative morbidity and mortality for surgical patients with pulmonary TB.

There are several possible explanations for the association between pulmonary TB and perioperative adverse events. First, pulmonary TB infection induces pulmonary inflammation and alters innate immunity in ways that may stimulate lung remodeling, impaired pulmonary function, pneumonia, and sepsis [38]. This may partly explain increased postoperative septicemia and pneumonia in patients with pulmonary TB. Second, complex regimens of anti-TB drugs may influence postoperative adverse events. For example, interactions between anti-TB drugs and side effects such as hepatic, ocular, and renal toxicity might contribute to negative postoperative outcomes in surgical patients with pulmonary TB [39,40]. Third, patients with pulmonary TB often are members of groups with lower socioeconomic status [41]. Poor living environments and difficulty accessing medical care (leading to delayed diagnosis and treatment for TB) increase the severity of disease and subsequent postoperative mortality [42]. Fourth, patients with pulmonary TB may develop extrapulmonary TB and opportunistic infections because of decreased immune function [8,43], which also may result in more infectious illness after major surgeries. In addition, because extrapulmonary TB is atypical, it may not be considered in the initial diagnosis of pulmonary TB. Experienced physicians should obtain appropriate specimens from patients with possible extrapulmonary tuberculosis for acid-fast bacillus stain, mycobacterial culture, and histology [30,43]. Confirmation of extrapulmonary TB is difficult, so this may be poorly recognized and misdiagnosed, especially when the disease may involve unusual sites. This may be a reason why higher postoperative mortality was found in patients with extrapulmonary TB. Finally, patient compliance with six months of treatment remains a problem in TB management [44]. Poor adherence and loss to follow-up lead to aggravation of pulmonary TB and other co-infections [45] that increase postoperative adverse outcomes.

One limitation of our study was a lack of detailed information on patients’ lifestyle habits and on biochemical examinations. The National Health Insurance Research Database does not include test results and information concerning chest X-rays, sputum cultures, nutritional status, and severity of disease. The second limitation would be cases where patients without symptoms are undiagnosed or not officially reported, although pulmonary TB is covered in national surveillance efforts. These undiagnosed or unreported pulmonary TB cases may be classified into the non-TB group that causes possible underestimation of the impact of pulmonary TB on postoperative adverse events.

Our study suggests that surgical patients with pulmonary TB faced higher risks of postoperative complications and mortality than patients without pulmonary TB. These findings highlight the complex issues involved in providing care for patients with pulmonary TB who receive non-chest surgeries. To better serve this group, it is crucial to create awareness about such negative outcomes’ causes and prevention, as well as using this information to provide optimal surgical services. These research results can help medical teams to provide proper preoperative and postoperative management to reduce adverse outcomes in this population.
