# Correction: Adverse Outcomes after Non-Chest Surgeries in Patients with Pulmonary Tuberculosis: A Nationwide Study

**DOI:** 10.1371/journal.pone.0175603

**Published:** 2017-04-06

**Authors:** Chi-Chen Ke, Chao-Shun Lin, Chun-Chieh Yeh, Chi-Li Chung, Chih-Jen Hung, Chien-Chang Liao, Ta-Liang Chen

There is an error in affiliation 4 for co-authors Chao-Shun Lin, Chien-Chang Liao, and Ta-Liang Chen. Affiliation 4 should be: Department of Anesthesiology, School of Medicine, College of Medicine, Taipei Medical University, Taipei, Taiwan.
